# Physical activity, dietary habits, screen time, and sleep duration among Saudi male adolescent athletes and non-athletes: a cross-sectional study

**DOI:** 10.3389/fpubh.2026.1871762

**Published:** 2026-06-24

**Authors:** Fahad Bin Radhyan, Rakesh Tomar, Abdulmalek K. Bursais, Abdulrahman I. Alaqil

**Affiliations:** 1 Department of Physical Education, College of General Studies, King Fahd University of Petroleum and Minerals, Dhahran, Saudi Arabia; 2 Department of Physical Education, College of Education, King Faisal University, Al-Ahsa, Saudi Arabia

**Keywords:** adolescents, body mass index, dietary habits, physical activity, Saudi Arabia, sedentary behavior, sleep duration

## Abstract

**Background:**

Adolescence represents a pivotal stage for the development of lifelong health behaviors. In Saudi Arabia, rising obesity and sedentary lifestyles among youth represent growing public health challenges. While organized sports may positively influence lifestyle behaviors, evidence comparing Saudi adolescent athletes and non-athletes across multiple behavioral domains remains limited.

**Purpose:**

To compare lifestyle habits, including physical activity levels, sedentary behaviors, dietary practices, and sleep duration, between Saudi male adolescent athletes and non-athletes.

**Method:**

A cross-sectional study of 124 male high school students aged 16–17 years (70 athletes, 54 non-athletes) from Riyadh used a validated 47-item Arab Teen Lifestyle Study (ATLS) questionnaire. Physical activity was measured in METs-minutes per week. Chi-square tests, independent *t*-tests, and multiple linear regression were applied.

**Result:**

Athletes exhibited significantly higher physical activity levels (98.6% meeting guidelines compared to 55.6% of non-athletes; *p* < 0.001) and lower BMI (23.64 ± 5.39 versus 29.69 ± 8.34 kg/m^2^; *p* < 0.001). Additionally, a greater proportion of athletes reported consuming fruit on five or more days per week (54.3% versus 33.3%; χ^2^ = 8.10, *p* = 0.017; V = 0.26). Athletes reported shorter average sleep duration (9.06 ± 3.67 versus 11.28 ± 3.52 h per day; *p* = 0.001), although both groups exceeded the minimum recommended 8 h. No significant difference was observed between groups in daily screen time (4.04 ± 2.63 versus 4.44 ± 3.08 h; *p* = 0.44). In an exploratory regression model, frequency of energy drink consumption emerged as the only significant individual predictor of BMI (*B* = 4.12, 95% CI: 0.32–7.92; *p* = 0.034), although the overall model was not statistically significant (*R*^2^ = 0.071; *F* = 1.26; *p* = 0.278).

**Conclusion:**

Participation in organized sports is linked to increased physical activity, lower body mass index, and improved fruit consumption among Saudi male adolescents. However, athletes reported reduced sleep duration, and elevated screen time persisted in both groups. These results support the integration of sports programs into adolescent health strategies and highlight the need for comprehensive interventions that address sleep hygiene, screen time management, and the promotion of physical activity.

## 1 Introduction

Establishing healthy lifestyle behaviors during adolescence is critical for public health, as early-life patterns of physical activity, sedentary behavior, dietary habits, and sleep often persist into adulthood and influence long-term cardiometabolic and chronic disease risk (1–7). Although the World Health Organization (WHO) recommends that adolescents engage in an average of 60 min of moderate-to-vigorous physical activity daily, adherence to these guidelines remains low, and inactivity rates are rising globally (8, 9). This issue is particularly acute in the Eastern Mediterranean region, where population-based studies report that over 60% of adolescents do not meet the recommended daily physical activity levels (1, 9, 10).

In addition to insufficient physical activity, sedentary behaviors, particularly recreational screen time, have been identified as independent risk factors for obesity, cardiometabolic dysregulation, and poor mental health among adolescents (11–16). Adolescent dietary patterns are also problematic; studies in Arab populations report high consumption of sugar-sweetened beverages and fast food, low intake of fruits and vegetables, and irregular breakfast habits (1, 17). Furthermore, sleep duration is an often-overlooked lifestyle factor, with insufficient sleep during adolescence associated with impaired cognitive function, metabolic dysregulation, and increased risk of obesity (18, 19).

Saudi Arabia provides a distinctive context for examining adolescent lifestyle behaviors, given its rapid urbanization, socioeconomic development, and significant lifestyle transitions over recent decades (1, 20, 21). Data from the Arab Teens Lifestyle Study (ATLS), the primary multi-site surveillance tool for Saudi adolescent health behaviors, indicate that 60–71% of Saudi adolescents do not meet physical activity recommendations. High rates of sedentary behavior, unhealthy dietary habits, and short sleep duration have also been consistently documented (22, 23). Comparable patterns have been observed across neighboring Gulf Cooperation Council (GCC) countries (17), highlighting a regional public health challenge.

Participation in organized sports has been identified as a factor that promotes healthier lifestyles among adolescents, with athletes often exhibiting more favorable behavioral profiles than their non-athlete peers across multiple domains (24–26). However, there is limited evidence directly comparing Saudi adolescent athletes and non-athletes across a comprehensive range of lifestyle behaviors, including physical activity, sedentary behavior, dietary habits, and sleep duration. Such comparative data are crucial for developing targeted, evidence-based interventions and informing sports policy within the Saudi context. To address this gap, the present study compares lifestyle habits, specifically physical activity levels, sedentary behaviors, dietary practices, and sleep duration, between Saudi male adolescent athletes and non-athletes.

## 2 Method

### 2.1 Study design and setting

This cross-sectional study was conducted in Riyadh, Saudi Arabia. Ethical approval was obtained from the Institutional Review Board of King Saud University (Reference number: KSU-HE-22-891). All procedures adhered to the principles of the Declaration of Helsinki. Written informed consent was obtained from all participants. As participants were aged 11–17 years, written parental or guardian assent was also obtained prior to enrolment.

### 2.2 Participants and recruitment

Participants were recruited from male-only public high schools in Riyadh using convenience sampling. Athletes (*n* = 70) were defined as students who were active members of school or community sports club teams and trained at least three sessions per week in any sport discipline. Non-athletes (*n* = 54) were students from the same schools who did not participate in any organized sport or structured physical training program. Individuals with known medical conditions affecting physical activity or dietary behavior were excluded. The final sample included 124 male adolescents aged 16–17 years. *A priori* power analysis using G*Power (version 3.1.9.7) determined that a sample size of 124 participants was adequate to detect a medium effect size (*d* = 0.50) with 80% power at a two-tailed significance level of *α* = 0.05 for an independent samples *t*-test (27).

### 2.3 Anthropometric measurements

Standing height was measured to the nearest 0.1 cm using a calibrated stadiometer (Deteco Electronic, Model: DHRWM, USA), and weight was recorded to the nearest 0.1 kg using a digital scale (Seca-869, Germany). Body mass index (BMI) was calculated as body mass in kilograms divided by height squared in meters (kg/m2). Waist circumference (WC) was measured twice at the midpoint between the lowest rib and the iliac crest using a non-stretchable tape measure (Body Care, Germany), and the average of the two measurements was used in the analysis (27).

### 2.4 Study questionnaire

Data on lifestyle variables were collected using the ATLS questionnaire, a validated, self-administered instrument consisting of 47 items designed to evaluate health-related behaviors among adolescents and young adults aged 14–25 years (18). The ATLS has demonstrated acceptable reliability and validity for measuring physical activity, sedentary behavior, sleep duration, and dietary habits in the Arab adolescent population.

### 2.5 Physical activity assessment

Physical activity data were collected using the ATLS questionnaire: the frequency, duration, and perceived intensity of activities performed during a typical week across domains such as walking, moderate-intensity activity, vigorous-intensity activity, and household chores. Activities were categorized by intensity level as light, moderate, or vigorous. Total weekly physical activity energy expenditure was calculated in METs-minutes per week (METs-min/week). Participants were considered physically active if they accumulated at least 1,680 METs-min/week, corresponding to the WHO recommendation of 60 min of moderate-to-vigorous physical activity (MVPA) per day (8).

### 2.6 Sedentary behaviors and sleep duration

Sedentary behaviors were defined as total daily recreational screen time, including television viewing, video gaming, computer use, and internet browsing on both weekdays and weekends. Participants were categorized as exceeding recommendations if their mean daily screen time was >2 h, in accordance with widely accepted pediatric guidelines (28). Sleep duration was measured separately for weekdays and weekends; mean sleep duration was calculated as a weighted average across both. Participants were classified as obtaining sufficient sleep if their mean daily sleep duration was >8 h (19).

### 2.7 Dietary habits

Dietary behavior data were gathered using the ATLS questionnaire, specifically, the frequency with which 11 specific food and beverage items were consumed during a typical week: breakfast, vegetables, fruits, dairy products, sugar-sweetened beverages, fast food, fried potatoes, bakery products, sweets, and energy drinks. Response options ranged from 0 (never) to 7 days per week (daily) and were subsequently categorized into three groups for chi-square analysis: 1–2 days per week, 3–4 days per week, and ≥5 days per week.

### 2.8 Statistical analysis

IBM SPSS Software (version 21.0, IBM Corp., Armonk, NY, USA) was utilized for statistical analyses. Continuous variables are presented as mean ± standard deviation (SD), and categorical variables as frequency and percentage (%) with 95% confidence intervals (95% CI). Between-group differences for continuous variables were assessed using independent samples *t*-tests, with effect sizes reported as Cohen’s *d*. Associations between athlete status and categorical lifestyle variables were evaluated using Pearson chi-square tests, and effect sizes were reported as Cramer’s *V* (small ≈ 0.10, medium ≈ 0.30, large ≈ 0.50) (29). Due to multiple chi-square comparisons across 11 dietary and behavioral variables, a Bonferroni-corrected significance threshold of *p* < 0.005 was applied. Associations significant at *p* < 0.05 but not meeting the corrected threshold are described as nominally significant. An exploratory multiple linear regression analysis was performed to identify predictors of BMI, with lifestyle and dietary variables included as independent variables. Results are presented as unstandardized (B) and standardized (*β*) coefficients with 95% CIs. A two-tailed *α* of 0.05 was considered the primary significance threshold for all other analyses.

## 3 Results

The study included 124 male adolescents, comprising 70 athletes and 54 non-athletes. The mean age of participants was 16.64 ± 0.63 years, with no significant difference between groups (16.66 ± 0.63 vs. 16.61 ± 0.63 years; *p* = 0.688). Compared to non-athletes, athletes exhibited significantly lower body weight (72.56 ± 17.35 vs. 91.04 ± 22.71 kg; *p* < 0.001; *d* = 0.93), lower body mass index (BMI) (23.64 ± 5.39 vs. 29.69 ± 8.34 kg/m^2^; *p* < 0.001; *d* = 0.89), and smaller waist circumference (85.46 ± 12.61 vs. 95.50 ± 17.89 cm; *p* = 0.001; *d* = 0.67) as shown in Table 1.

**Table 1:** Descriptive statistics of anthropometric and physical activity variables (Mean ± SD) by group.

Variable	Overall *N* = 124	Non-athletes *N* = 54	Athletes *N* = 70
Age (years)	16.64 (0.63)	16.6 (0.63)	16.66 (0.63)
Weight (kg)	80.60 (21.81)	91.04 (22.71)	72.56 (17.35)
Height (m)	1.74 (0.061)	1.74 (0.07)	1.75 (0.05)
BMI (kg/m^2^)	26.27 (7.44)	29.69 (8.34)	23.64 (5.39)
Waist circumference (cm)	89.83 (15.87)	95.50 (17.89)	85.46 (12.61)
Walking (METs-min/week)	573.32 (533.11)	398.27 (471.52)	708.35 (541.64)
Moderate sports activity (METs-min/week)	494.13 (594.69)	325.18 (505.19)	624.46 (628.35)
Vigorous sports activity (METs-min/week)	849.35 (723.57)	520.00 (692.17)	1103.43 (644.13)
Household activity (METs-min/week)	265.83 (404.47)	165.81 (260.90)	342.99 (474.85)
Total MET (min/week)	2182.63 (1600.70)	1409.27 (1478.82)	2779.22 (1434.75)
Screen time (hours/day)	4.22 (2.83)	4.44 (3.08)	4.04 (2.63)
Sleep time (hours/day)	10.02 (3.75)	11.28 (3.52)	9.06 (3.67)

Data are presented as mean (standard deviation). BMI = body mass index; MET = metabolic equivalent of task.

Athletes exhibited significantly higher levels of physical activity across all measurement domains (Tables 1, 2). Total metabolic equivalent (MET) scores were substantially greater among athletes (2,779.22 ± 1,434.75 vs. 1,409.27 ± 1,478.82 METs-min/week; *p* < 0.001; *d* = 0.94), and a markedly higher proportion of athletes met established physical activity guidelines (98.6% vs. 55.6%; Table 3). No significant difference was observed between groups in mean daily screen time (athletes: 4.04 ± 2.63 vs. non-athletes: 4.44 ± 3.08 h/day; *p* = 0.436; *d* = 0.14), with more than 60% of the total sample exceeding the recommended 2-h daily threshold (Tables 1–3).

**Table 2:** Descriptive statistics of dietary behaviors with 95% confidence intervals among athletes and non-athletes (*N* = 124).

Variable	Non-athletes *N* = 54 (%)	Athletes *N* = 70 (%)	Overall *N* = 124 (%)	95% CI
Breakfast frequency				
1–2 days/week	15 (27.8)	24 (34.3)	39 (31.5)	23.9–40.1
3–4 days/week	4 (7.4)	9 (12.9)	13 (10.5)	6.2–17.1
≥5 days/week	35 (64.8)	37 (52.9)	72 (58.1)	49.3–66.4
Sugary beverage consumption				
1–2 days/week	26 (48.1)	34 (48.6)	60 (48.4)	39.8–57.1
3–4 days/week	18 (33.3)	15 (21.4)	33 (26.6)	19.6–35.0
≥5 days/week	10 (18.5)	21 (30)	31 (25.0)	18.2–33.3
Vegetables intake				
1–2 days/week	14 (25.9)	14 (20)	28 (22.6)	16.1–30.7
3–4 days/week	15 (27.8)	12 (17.1)	27 (21.8)	15.4–29.8
≥ 5 days/week	25 (46.3)	44 (62.9)	69 (55.6)	46.9–64.1
Fruits intake				
1–2 days/week	26 (48.1)	17 (24.3)	43 (34.7)	26.9–43.4
3–4 days/week	10 (18.5)	15 (21.4)	25 (20.2)	14.0–28.1
≥5 days/week	18 (33.3)	38 (54.3)	56 (45.2)	36.7–53.9
Dairy products intake				
1–2 days/week	12 (22.2)	16 (22.9)	28 (22.6)	16.1–30.7
3–4 days/week	18 (33.3)	13 (18.6)	31 (25.0)	18.2–33.3
≥ 5 days/week	24 (44.4)	41 (58.6)	65 (52.4)	43.7–61.0
Fast food intake				
1–2 days/week	32 (59.3)	38 (54.3)	70 (56.5)	47.7–64.9
3–4 days/week	14 (25.9)	16 (22.9)	30 (24.2)	17.5–32.4
≥5 days/week	8 (14.8)	16 (22.9)	24 (19.4)	13.4–27.2
Fried potatoes intake				
1–2 days/week	35 (64.8)	40 (57.1)	75 (60.5)	51.7–68.6
3–4 days/week	13 (24.1)	19 (27.1)	32 (25.8)	18.9–34.2
≥5 days/week	6 (11.1)	11 (15.7)	17 (13.7)	8.7–20.9
Bakery products consumption				
1–2 days/week	38 (70.4)	45 (64.3)	83 (66.9)	58.3–74.6
3–4 days/week	6 (11.1)	17 (24.3)	23 (18.5)	12.7–26.3
≥5 days/week	10 (18.5)	8 (11.4)	18 (14.5)	9.4–21.8
Sweets intake				
1–2 days/week	32 (59.3)	43 (61.4)	75 (60.5)	51.7–68.6
3–4 days/week	13 (24.1)	18 (25.7)	31 (25.0)	18.2–33.3
≥5 days/week	9 (16.7)	9 (12.9)	18 (14.5)	9.4–21.8
Energy drinks intake				
1–2 days/week	49 (90.7)	63 (90.0)	112 (90.3)	83.8–94.4
3–4 days/week	4 (7.4)	6 (8.6)	10 (8.1)	4.4–14.2
≥5 days/week	1 (1.9)	1 (1.4)	2 (1.6)	0.4–5.7

Data are presented as frequency (percentage). 95% CI applies to the Overall column. PA = physical activity.

**Table 3:** Descriptive statistics of lifestyle behaviors with 95% confidence intervals among athletes and non-athletes (*N* = 124).

Variable	Non-athletes *N* = 54 (%)	Athletes *N* = 70 (%)	Overall *N* = 124 (%)	95% CI
Physical activity status				
Inactive	39 (72.2)	18 (25.7)	57 (46)	37.4–54.7
Moderately active	4 (7.4)	17 (24.3)	21 (16.9)	11.4–24.5
Highly active	11 (20.4)	35 (50.0)	46 (37.1)	29.1–45.9
Screen time				
≤2 h.	18 (33.3)	30 (42.9)	48 (38.7)	30.6–47.5
>2 h.	36 (66.7)	40 (57.1)	76 (61.3)	52.5–69.4
Sleeping time				
≤8 h.	10 (18.5)	32 (45.7)	42 (33.9)	26.1–42.6
>8 h.	44 (81.5)	38 (54.3)	82 (66.1)	57.4–73.9

Chi-square analysis indicated a nominally significant relationship between athlete status and fruit intake (χ^2^ = 8.10, *p* = 0.017; *V* = 0.26), with athletes demonstrating higher rates of adequate fruit consumption (≥5 days/week: 54.3% compared to 33.3%; Table 4). However, this association did not remain significant after correction for multiple comparisons (corrected threshold *p* < 0.005). No statistically significant associations were observed for breakfast frequency, vegetable intake, dairy product consumption, sugar-sweetened beverage intake, fast food, fried potato, bakery products, sweets, or energy drink consumption (all *p* > 0.05; Table 4).

**Table 4:** Chi-square analysis of associations between athlete status and lifestyle and dietary behaviors.

Variable	Non-athlete *N* = 54 (%)	Athlete *N* = 70 (%)	χ^2^	*p-*value	Effect size
Screen time			1.165	0.353	0.097
≤2 h.	18 (33.3)	36 (66.7)			
>2 h.	26 (42.9)	40 (57.1)			
Sleep time			10.066	0.002*	0.285
≤8 h.	10 (18.5)	32 (45.7)			
>8 h.	44 (81.5)	38 (54.3)			
Breakfast frequency			2.025	0.363	0.128
Irregular	15 (27.8)	24 (34.3)			
Occasional	4 (7.4)	9 (12.9)			
Regular	35 (64.8)	37 (52.9)			
Sugary beverage consumption			3.232	0.199	0.161
Low consumption	26 (48.1)	34 (48.6)			
Moderate consumption	18 (33.3)	15 (21.4)			
High consumption	10 (18.5)	21 (30.0)			
Vegetable intake			3.560	0.169	1.69
Low intake	14 (25.9)	14 (20.0)			
Moderate intake	15 (27.8)	12 (17.1)			
Adequate intake	25 (46.3)	44 (62.9)			
Fruit intake			8.097	0.017*	0.256
Low intake	26 (48.1%)	17 (24.3)			
Moderate intake	10 (18.5)	15 (21.4)			
Adequate intake	18 (33.3)	38 (54.3)			
Dairy product intake			3.823	0.148	0.176
Low intake	12 (22.2)	16 (22.9)			
Moderate intake	18 (33.3)	13 (18.6)			
Adequate intake	24 (44.4)	41 (58.6)			
Fast food intake			1.271	0.530	0.101
Low consumption	32 (59.3)	38 (54.3)			
Moderate consumption	14 (25.9)	16 (22.9)			
High consumption	8 (14.8)	16 (22.9)			
Fried potato intake			0.879	0.644	0.084
Low consumption	35 (64.8)	40 (57.1)			
Moderate consumption	13 (24.1)	19 (27.1)			
High consumption	6 (11.1)	11 (15.7)			
Bakery product consumption			4.077	0.130	0.181
Low consumption	38 (70.4)	45 (64.3)			
Moderate consumption	6 (11.1)	17 (24.3)			
High consumption	10 (18.5)	8 (11.4)			
Sweet intake			0.361	0.835	0.054
Low consumption	32 (59.3)	43 (61.4)			
Moderate consumption	13 (24.1)	18 (25.7)			
High consumption	9 (16.7)	9 (12.9)			
Energy drink intake			0.087	0.957	0.026
Low consumption	49 (90.7)	63 (90.0)			
Moderate consumption	4 (7.4)	6 (8.6)			
High consumption	1 (1.9)	1 (1.6)			

**p < 0.005 (Bonferroni-corrected threshold for 11 comparisons). *p < 0.05 (nominally significant only). Cramer’s *V*: small ≈ 0.10, medium ≈ 0.30, large ≈ 0.50.

Athletes demonstrated a significantly shorter mean daily sleep duration compared to non-athletes (9.06 ± 3.67 vs. 11.28 ± 3.52 h per day; *p* = 0.001; *d* = 0.62). Additionally, a greater proportion of athletes slept 8 h or less per day (45.7% vs. 18.5%; χ^2^ = 10.07, *p* = 0.002; *V* = 0.29). However, both groups exceeded the minimum recommended 8 h of sleep per night (Tables 1, 4, 5).

**Table 5:** Between-group comparison of anthropometric and physical activity variables using independent samples *t*-test.

Variable	Non-athlete *N* = 54 mean (SD)	Athlete *N* = 70 mean (SD)	*p-*value	Cohen’s *d*	Confidence interval
Age (years)	16.611 (0.626)	16.657 (0.634)	0.688	−0.07	−0.272 to 0.180
Weight (kg)	91.037 (22.705)	72.557 (17.350)	0.000**	0.93	11.360 to 25.598
Height (m)	1.735 (0.0694)	1.749 (0.053)	0.155	−0.26	−0.038 to 0.006
BMI (kg/m^2^)	29.691 (8.337)	23.636 (5.389)	0.000**	0.89	3.607 to 8.503
Waist circumference (cm)	95.500 (17.886)	85.457 (12.607)	0.001*	0.67	4.618 to 15.467
Walking (METs-min/week)	398.272 (471.523)	708.350 (541.638)	0.001*	−0.61	−493.780 to −126.374
Moderate sports activity (METs-min/week)	325.185 (505.190)	624.457 (628.348)	0.004*	−0.52	−506.538 to −92.005
Vigorous sports activity (METs-min/week)	520.000 (692.166)	1103.428 (644.134)	0.000**	−0.88	−822.013 to −344.843
Household activity (METs-min/week)	165.814 (260.901)	342.985 (474.845)	0.009*	−0.45	−319.280 to −35.060
Total MET (min/week)	1409.272 (1478.819)	2779.221 (1434.748)	0.000**	−0.95	−1891.292 to −848.605
Screen time (hours/day)	4.444 (3.075)	4.042 (2.634)	0.436	0.14	−0.614 to 1.417
Sleeping hours (hours/day)	11.277 (3.515)	9.057 (3.666)	0.001*	0.62	0.929 to 3.512

*p < 0.05, **p < 0.001, Cohen’s *d*: small = 0.20, medium = 0.50, large = 0.80.

**Figure 1:**
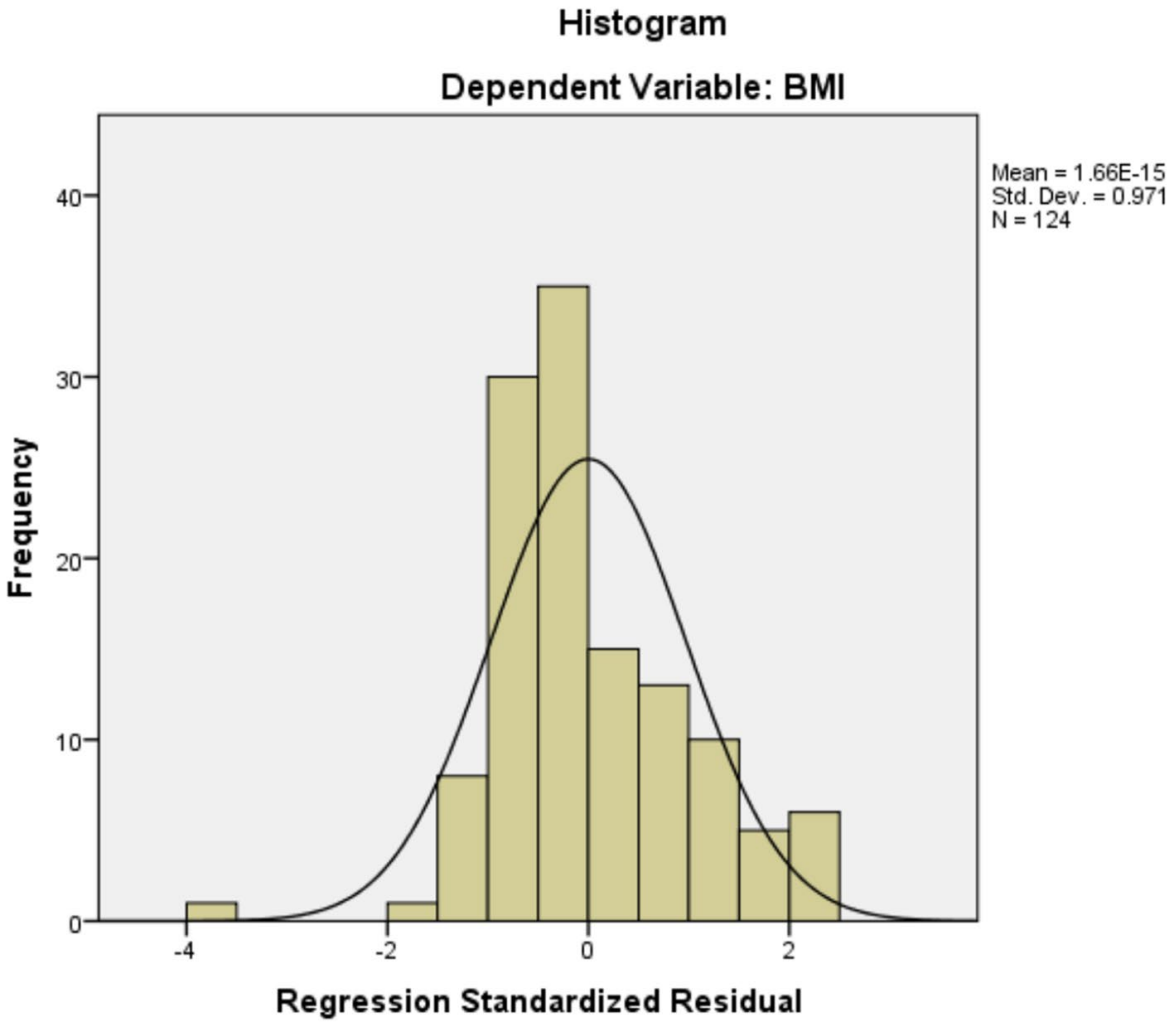
Histogram of residuals.

**Figure 2:**
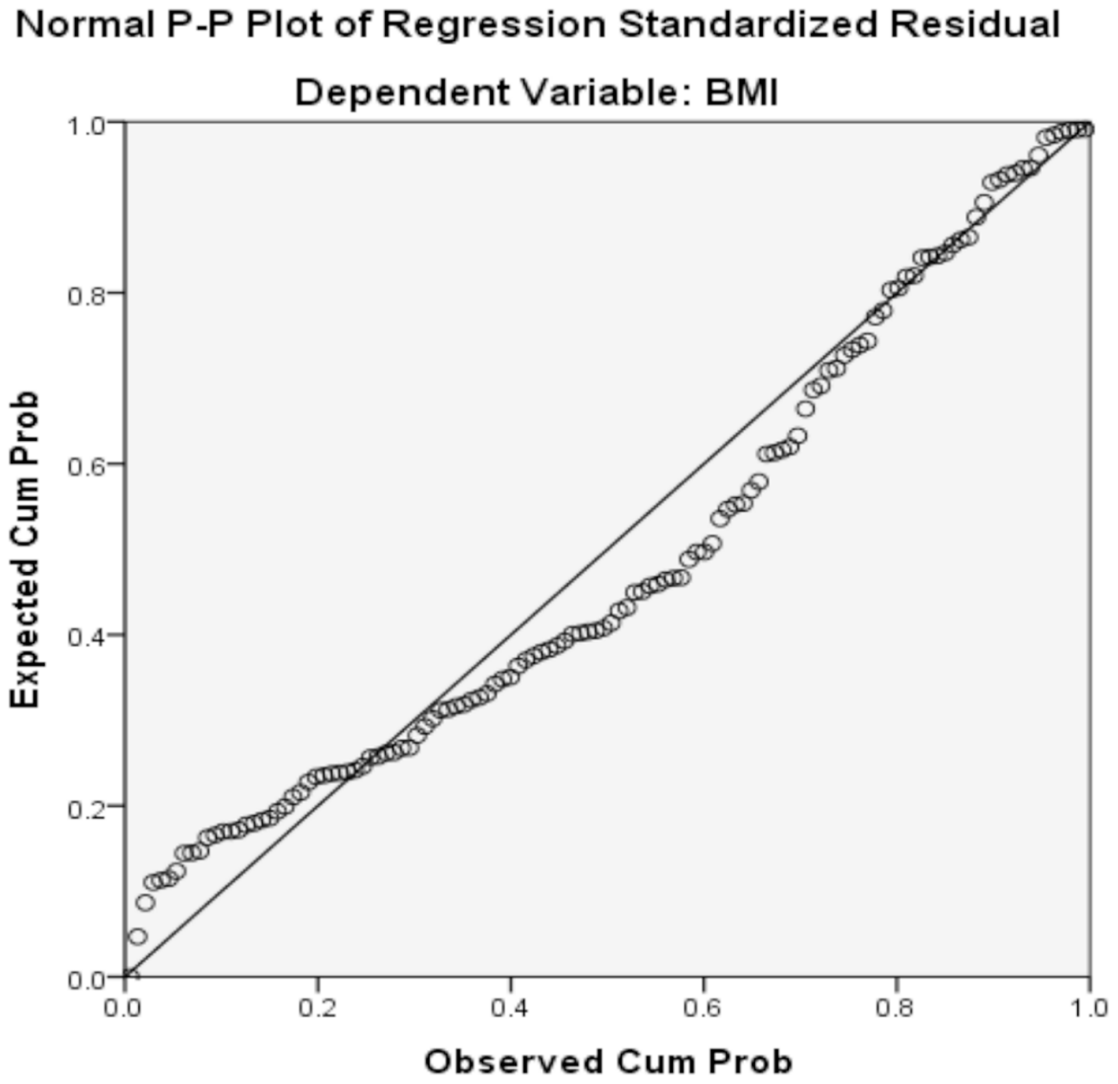
Normal P–P Plot.

**Figure 3:**
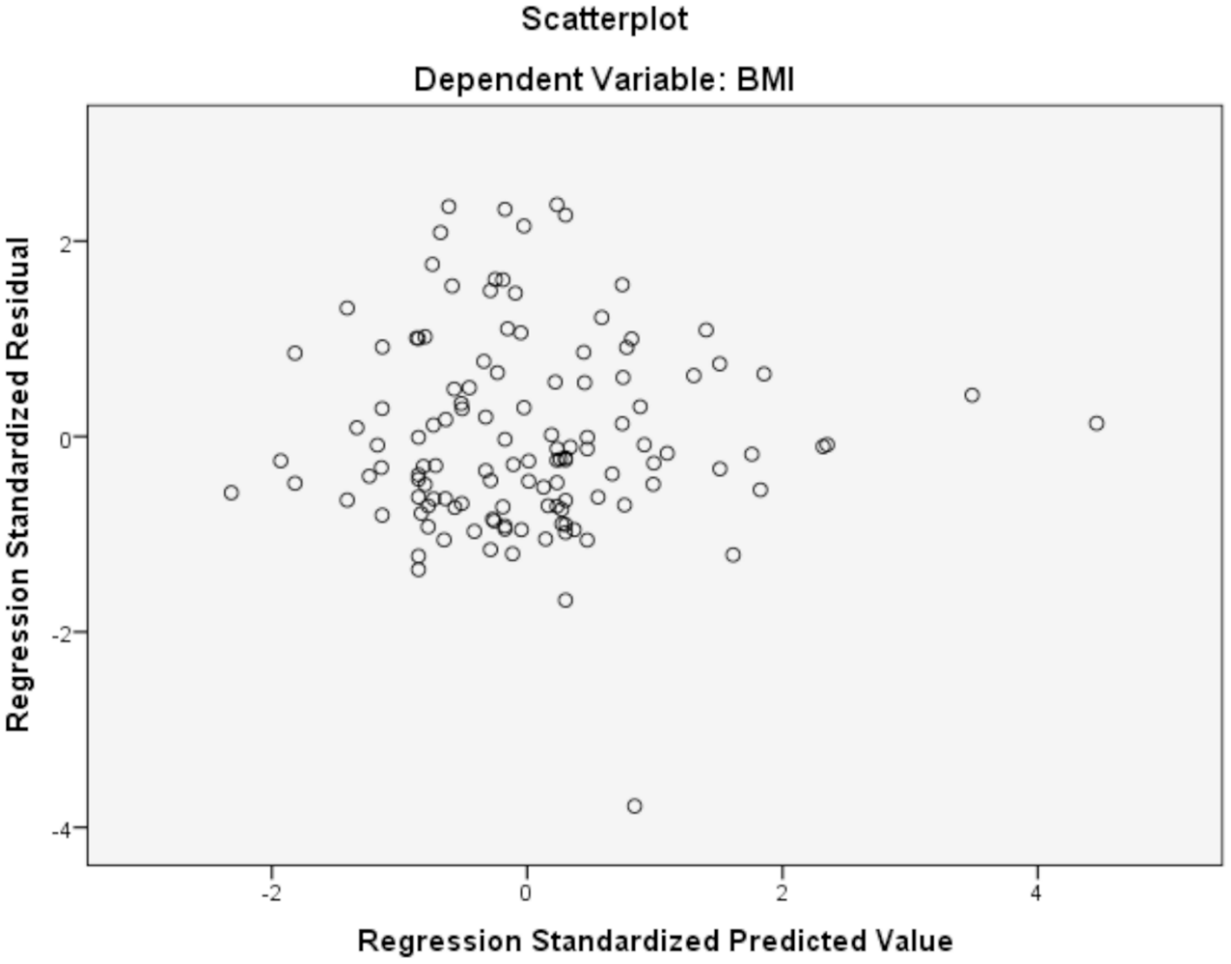
The residual scatterplot.

Prior to conducting multiple regression analysis, assumptions were examined. Multicollinearity was assessed using tolerance and Variance Inflation Factor (VIF) statistics. All tolerance values exceeded 0.10 and VIF values were below 5, indicating no multicollinearity issues. Residuals were approximately normally distributed based on histogram and P–P plot inspection. The Durbin–Watson statistic (1.389) indicated independence of errors. Furthermore, the scatterplot of standardized residuals versus standardized predicted values demonstrated homoscedasticity (Figures 1–3).

An exploratory multiple linear regression model including physical activity status, breakfast frequency, vegetable intake, fast food, bakery products, sweets, and energy drink consumption as predictors of BMI did not reach statistical significance overall [*R*^2^ = 0.071, *F* (7,116) = 1.26, *p* = 0.278], explaining only 7.1% of the variance in BMI. Because the omnibus test was non-significant, individual coefficients within this model cannot be interpreted as established predictors. Energy drink consumption frequency reached a nominal *p*-value within the model (*B* = 4.12, *β* = 0.20, 95% CI: 0.32–7.92; *p* = 0.034; Table 6), but this result is reported for descriptive purposes only and should be regarded as a hypothesis-generating signal rather than evidence of an independent predictor of BMI.

**Table 6:** Exploratory multiple linear regression analysis: predictors of body mass index (BMI).

Model	*B*	Std. error	Beta (β)	*P*-value	95.0% Confidence interval for B	Collinearity statistics	
						Tolerance	VIF
(Constant)	21.889	3.734		0.000**	14.493–29.285		
Physical activity level	−1.071	0.749	−0.131	0.156	−2.555 - 0.413	0.952	1.050
Breakfast frequency	0.552	0.747	0.068	0.462	−0.927–2.030	0.957	1.045
Vegetables intake	−0.133	0.826	−0.015	0.873	−1.769 – 1.504	0.958	1.044
Fast food intake	0.075	0.886	0.008	0.933	−1.680 – 1.829	0.904	1.106
Bakery product consumption	−1.030	1.036	−0.102	0.322	−3.082 – 1.022	0.759	1.317
Sweet intake	1.498	0.999	0.148	0.137	−0.481–3.478	0.817	1.223
Energy drink intake	4.117	1.918	0.202	0.034*	0.318–7.916	0.903	1.108

Model summary: *R*^2^ = 0.071, *F*(7, 116) = 1.26, *p* = 0.278. The overall model was not statistically significant; individual predictor estimates should be interpreted with caution. * Nominally significant at *p* < 0.05 within a non-significant omnibus model; this association is exploratory and requires confirmation in future studies. **p < 0.001. Tolerance > 0.10, VIF < 5.

## 4 Discussion

This study compared Saudi male adolescent athletes and non-athletes on physical activity, sedentary behaviors, dietary habits, and sleep duration using the validated ATLS questionnaire. The principal findings were as follows: (i) athletes exhibited significantly higher physical activity levels and lower body mass index (BMI) than non-athletes; (ii) athletes reported slightly greater fruit consumption; (iii) although athletes participated in organized sports, they reported shorter sleep duration than non-athletes, yet both groups exceeded the minimum recommended threshold; and (iv) screen time did not differ significantly between groups and remained elevated in both. These results indicate that while organized sport participation is associated with some healthier behaviors, it does not uniformly promote a healthier lifestyle.

Substantial between-group differences in physical activity (*d* = 0.94) and BMI (*d* = 0.89) are consistent with prior evidence reporting associations between organized sport participation and more favorable profiles of energy expenditure and body composition during adolescence (26, 30–32). Because the present design is cross-sectional, these differences should be interpreted as associations rather than as causal effects of sport participation; self-selection bias, in which adolescents with lower adiposity or more favorable lifestyle profiles are more likely to enter and remain in organized sport, cannot be ruled out. With this caveat, the observed BMI difference (6.05 kg/m^2^) is noteworthy in Saudi Arabia, where adolescent obesity rates have increased markedly in recent decades (1, 33), and supports the broader public health rationale for expanding access to organized sports programs as one component of childhood obesity prevention strategies in the GCC region (34, 35).

The chi-square comparison of fruit intake yielded a nominal between-group difference (*V* = 0.26, *p* = 0.017) that did not survive the Bonferroni-corrected threshold (*p* < 0.005) and should therefore be treated as a preliminary, hypothesis-generating observation rather than as a robust between-group difference. With this caveat, the direction of the effect is broadly consistent with Heikkilä et al. (2021), who reported higher fruit and vegetable intake among adolescent sports club participants (36). If replicated in adequately powered samples, such a pattern could plausibly reflect peer influence, coaching guidance, or increased nutritional awareness within the sports environment. Importantly, no nominal differences were detected between athletes and non-athletes for fast food, sugar-sweetened beverages, fried potatoes, bakery products, sweets, or energy drinks, indicating that sport participation alone is unlikely to drive comprehensive dietary improvement. Embedding structured nutrition education within sports programs may therefore be needed to address broader dietary patterns rather than isolated food groups.

The observation that athletes reported significantly shorter sleep duration than non-athletes (*d* = 0.62), despite engaging in markedly higher levels of physical activity, requires careful interpretation. Although counterintuitive at first glance, this pattern is plausible when considered alongside the structure of adolescent athlete schedules. Training sessions for school and community sports clubs in Saudi Arabia are commonly held either early in the morning before school or late in the evening after academic obligations are completed; both arrangements compress the available sleep window by advancing wake time or delaying bedtime (37, 38). Late-evening training is, in addition, associated with elevated post-exercise core body temperature, sympathetic nervous system activation, and cortisol concentrations, all of which can delay sleep onset and reduce sleep efficiency. These scheduling pressures are compounded by academic demands, since adolescent athletes must balance training, competition, and travel with the same coursework as their non-athlete peers, leaving limited recovery time and contributing to weekday sleep restriction. The mean of 9.06 h per night observed in our athletes therefore exceeds the general adolescent minimum of 8 h (19) but falls short of the 8.5–10 h recommended for athletic populations, an interval considered necessary to support muscle repair, hormonal regulation, and cognitive and athletic performance (39). Chronic mild sleep restriction in young athletes has been linked to impaired glucose tolerance, blunted growth hormone and testosterone secretion, slowed reaction time, and elevated injury risk, so the gap between observed and recommended sleep duration in this group is unlikely to be trivial. The unusually high mean sleep duration reported by non-athletes (11.28 h per day) also warrants comment. This value is higher than typical adolescent estimates and most likely reflects a combination of weekend catch-up sleep being incorporated into the weighted weekly mean, recognized overestimation in adolescent self-reported sleep (40), and the absence of training-related scheduling constraints among non-athletes; it should not be interpreted as an indicator of consistently high-quality habitual sleep. Taken together, these considerations highlight the need for school-based sleep-hygiene interventions, scheduling adjustments to limit very early or very late training sessions, and coach- and parent-directed education on the role of sleep in recovery, all of which should be considered as components of comprehensive adolescent athlete health programs.

Elevated screen time persisted in both groups, with a mean exceeding 4 h per day and over 60% surpassing 2 h daily. This pattern is consistent with regional ATLS findings, which document widespread non-compliance with screen time guidelines among Saudi male adolescents (22). These results challenge the assumption that participation in organized sports reduces sedentary screen behavior. The simultaneous presence of high physical activity and high screen time among athletes supports the emerging consensus that these behaviors represent distinct and largely independent dimensions of adolescent health (13). Consequently, effective reduction of screen time will require targeted, multi-component strategies that address the environmental and social determinants of digital engagement, extending beyond the scope of physical activity interventions.

The present findings should also be interpreted within the broader context of Arab adolescent lifestyle research conducted using the ATLS framework and related methodologies across the Middle East and North Africa (MENA) region. Similar patterns of insufficient physical activity, prolonged screen time, and suboptimal dietary habits have been reported among adolescents in Kuwait, Qatar, and other Arab countries (17). In Kuwait, Allafi et al. reported high sedentary behavior and unhealthy dietary practices among adolescents, despite gender-related differences in physical activity participation (17). Likewise, regional reviews from the MENA region have consistently documented elevated screen time, low fruit and vegetable intake, and increasing obesity prevalence among adolescents, reflecting widespread lifestyle transitions associated with urbanization and digitalization (12). These similarities suggest that the behavioral patterns observed in Saudi adolescents are part of a broader regional public health challenge affecting Arab youth populations. Integrating findings from across the Arab region strengthens the external context of the present study and highlights the importance of culturally tailored, region-specific interventions targeting physical activity, nutrition, sleep hygiene, and sedentary behaviors among adolescents.

The exploratory multiple linear regression model did not reach overall statistical significance [*F*(7,116) = 1.26, *p* = 0.278, *R*^2^ = 0.071] and explained only 7.1% of the variance in BMI. When the omnibus test is non-significant, individual coefficients within the model are statistically unstable and cannot be treated as established predictors; accordingly, the nominal association observed for energy drink consumption (*B* = 4.12, *β* = 0.20, 95% CI: 0.32–7.92; *p* = 0.034) should be regarded as a hypothesis-generating signal rather than as evidence of an independent effect of energy drink intake on BMI. This caveat notwithstanding, the direction of the association is broadly consistent with literature linking frequent energy drink consumption to unfavorable metabolic and cardiovascular indicators in adolescents (41, 42). The low variance explained by the model further underscores that BMI in this population is shaped by a wide range of interacting biological, behavioral, and environmental factors not captured here. Targeted studies using larger samples, longitudinal designs, and objective dietary assessment are required before any independent or causal contribution of energy drink intake to adolescent BMI can be inferred.

Al Sabbah et al. reported significant associations between sedentary behavior, poor dietary habits, and overweight status among Palestinian adolescents (40). Likewise, Musaiger et al. demonstrated that adolescents across several Arab countries experience common barriers to healthy eating and regular physical activity, including environmental, social, and lifestyle-related constraints associated with rapid urbanization and technological transition (43).

These regional similarities suggest that the behavioral patterns identified in Saudi adolescents are not isolated phenomena but rather reflect a broader regional public health challenge affecting Arab youth populations. The rapid nutrition and lifestyle transition occurring across the Eastern Mediterranean region has been linked to increased consumption of energy-dense foods, reduced physical activity, and greater reliance on screen-based entertainment among adolescents (44, 45). Furthermore, systematic reviews from Gulf Cooperation Council (GCC) countries and the wider Arabian Peninsula continue to document high levels of physical inactivity and sedentary behavior despite growing awareness of the health consequences (46). The consistency of these findings across Arab populations strengthens the external relevance of the present study and highlights the need for culturally tailored, region-specific interventions that jointly address physical activity promotion, dietary improvement, sleep hygiene, and reduction of sedentary behaviors among adolescents.

## 5 Limitations

Several methodological limitations should be considered. The cross-sectional design prevents establishing causal relationships or determining the directionality of observed associations. Using a convenience sample from a single urban center in Riyadh may not accurately reflect Saudi adolescents in rural areas or other regions. We also emphasized the need for future studies using nationally representative, multi-region, and mixed-sex samples to improve the external validity of the findings. Reliance on self-reported data introduces potential recall and social desirability biases, particularly concerning dietary intake and physical activity (47). Treating athletes as a single group may have masked important behavioral differences between sports. The present analysis did not account for the heterogeneity of sports involvement, such as differences in sport type, training intensity, competitive level, and seasonal patterns, which may serve as important confounding factors. Future studies should include detailed sport participation profiles to better understand how different training environments and competitive demands influence adolescent health behaviors and outcomes. Future studies consider retaining continuous dietary variables or applying more advanced statistical modeling approaches to preserve greater analytical precision. Additionally, the sample was limited to males, restricting the generalizability of findings to female adolescents. Future research should utilize longitudinal designs, device-based measurement tools (such as accelerometry for physical activity and actigraphy for sleep assessment), multi-site sampling, and mixed-sex samples to address these limitations (48). Future studies should incorporate broader psychosocial and socioeconomic determinants to better understand the complex factors influencing adolescent health behaviors and body composition.

## 6 Conclusion

Organized sport participation was associated with significantly higher physical activity levels and lower BMI among Saudi male adolescents aged 16–17 years; a nominal association with higher fruit consumption was also observed but did not survive correction for multiple comparisons and requires replication. Athletes reported shorter sleep duration than non-athletes, although both groups exceeded the minimum eight-hour adolescent recommendation, and screen time was elevated and comparable across groups. The exploratory regression model was not statistically significant overall, and the nominal association observed between energy drink consumption and BMI should be regarded as a hypothesis-generating signal rather than evidence of an independent predictor. Because the design is cross-sectional, all findings should be interpreted as associations rather than as causal effects of sport participation. With these caveats in mind, the results support the inclusion of organized sport opportunities within broader adolescent health strategies in Saudi Arabia and the GCC region and reinforce the need for multi-component interventions that jointly address sleep hygiene, screen time, dietary patterns, and physical activity. Longitudinal studies employing objective measurement of physical activity, sleep, and diet are required to clarify the temporal and causal relationships among these behaviors.

## Data Availability

The raw data supporting the conclusions of this article will be made available by the authors, without undue reservation.
